# The ratio of HDL-C to apoA-I interacts with free triiodothyronine to modulate coronary artery disease risk

**DOI:** 10.1186/s12872-021-02316-8

**Published:** 2021-10-19

**Authors:** Li Li, Gaojun Cai, Wei Lu, Feng Li, Lei Yu, Jianqiang Xiao

**Affiliations:** Department of Cardiology, Wujin Hospital Affiliated to Jiangsu University, The Wujin Clinical College of XuZhou Medical University, Changzhou, 213017 Jiangsu China

**Keywords:** Coronary artery disease, Free triiodothyronine, High-density lipoprotein cholesterol/apolipoprotein A-I

## Abstract

**Objective:**

In the present work, research was carried out to explore the correlation between the high-density lipoprotein cholesterol (HDL-C)/apolipoprotein A-I (apoA-I) ratio and serum free triiodothyronine (FT3) and their interaction on the risk of coronary artery disease (CAD).

**Methods:**

A total of 1686 patients who underwent selective coronary angiography were enrolled in the present study, including 1279 patients with CAD and 407 controls. The subjects were divided into three groups according to tertiles of the HDL-C/apoA-I ratio. Binary logistic regression analysis was used to evaluate the interaction of the HDL-C/apoA-I ratio and FT3 level with the risk of CAD.

**Results:**

The group with the highest HDL-C/apoA-I ratio had the lowest levels of FT3. Multiple linear regression analysis showed that the HDL-C/apoA-I ratio was negatively associated with FT3 after adjusting for age, sex, body mass index (BMI), triglycerides (TGs), low-density lipoprotein cholesterol (LDL-C), apolipoprotein B (apoB), FT4 and TSH. A logistic regression model showed that a high HDL-C/apoA-I ratio (> 0.89 mmol/g) and high FT3 levels (> 4.5 pmol/l) were protective factors for CAD. Patients with a lower HDL-C/apoA-I ratio (≤ 0.89 mmol/g) and lower FT3 level (≤ 4.5 pmol/l) had an increased risk of CAD (OR = 2.441, *P* = 0.000, S = 1.13, AP = 0.068, AP* = 0.116, RERI = 0.168).

**Conclusions:**

The HDL-C/apoA-I ratio was negatively associated with FT3, and there was a significant interaction between the HDL-C/apoA-I ratio and FT3 with the risk of CAD.

## Introduction

High-density lipoprotein cholesterol (HDL-C) is a protective factor against coronary artery disease (CAD) [1]. However, some studies suggested that raising HDL-C levels, by high-dose niacin or inhibitors of cholesteryl ester transfer protein, may have no effect on vascular events including cardiovascular mortality and morbidity [2–4]. Apolipoprotein A-I (apoA-I) is the main protein constituent of HDL particles, that plays important atheroprotective functions as antioxidant, anti-inflammatory, antithrombotic, and nitric oxide-promoting properties, against CAD [5–7]. The HDL-C/apoA-I ratio was used to estimate HDL size in a previous study [8], and this lipid ratio could be more valuable than a single lipid level for predicting CAD [9].

It has long been known that thyroid hormones play an important role in regulating cardiac function, hepatic fatty acids, cholesterol synthesis and metabolism. Subclinical hypothyroidism is associated with increased CAD mortality, heart failure, and blood coagulation, as well as an increased risk of stroke [10, 11]. Previous studies have focused more on the relationship between thyroid hormones, thyroid stimulating hormone (TSH) and blood lipids, but thus far, the correlation between the HDL-C/apoA-I ratio and free triiodothyronine (FT3) has been less studied [12]. The purpose of our study was to explore the correlation between the HDL-C/apoA-I ratio and FT3 and their interaction with the risk of CAD.

## Materials and methods

### Study subjects

**Fig. 1:**
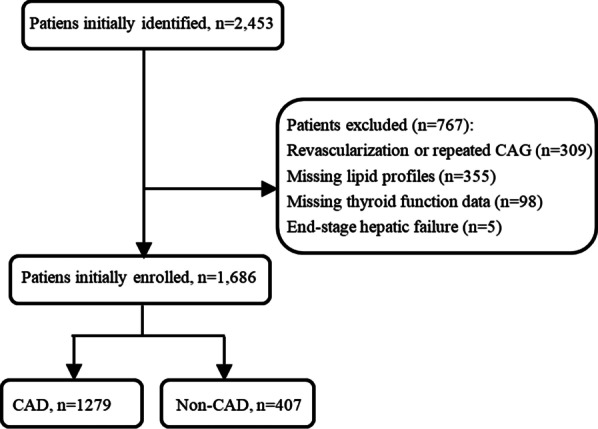
Flow of study participant selection

A total of 1686 patients (571 males and 1115 females) aged 29–95 years who underwent coronary angiography at Wujin Hospital affiliated with Jiangsu University were consecutively enrolled in this study between May 2017 and September 2019. The flowchart outlining the study is shown in Fig. 1. The exclusion criteria were as follows: patients who had undergone coronary revascularization or coronary angiography (CAG) examinations, participants with missing lipid profiles and thyroid function data, and end-stage hepatic failure. The study protocol was approved by the Ethics Committee of our hospital. This was a retrospective study, and informed consent could not be obtained from each patient, which was approved by the Ethics Committee of Wujin Hospital (No. 201610).

### Diagnostic criteria

CAD was defined in accordance with the 1979 WHO diagnostic criteria [13]. CAD was defined as a stenosis diameter greater than 50% in at least one major coronary vessel (left main, left anterior descending, left circumflex, right coronary artery, and large branches). All patients underwent a CAG examination. The CAG examinations were performed using the Judkin technique via the radial or femoral artery. Angiograms were analyzed by at least two experienced doctors who were blinded to this study. Control subjects were defined as those lacking typical angina pectoris symptoms and those in whom stenosis of the major coronary arteries was less than 50% [14].

Essential hypertension (EH) was defined as a systolic blood pressure (SBP) of more than 140 mmHg or diastolic blood pressure (DBP) of more than 90 mmHg on at least two occasions or individuals currently taking antihypertensive drugs [15]. Diabetes mellitus (DM) was diagnosed based on fasting plasma glucose ≥ 7.0 mmol/L and/or random glucose level ≥ 11.1 mmol/l, or with a medical diabetes record. A body mass index (BMI) < 28 kg/m^2^ was considered normal or overweight for adults and ≥ 28 kg/m^2^ considered obese [16].

### Laboratory and clinical measurements

Twelve-hour fasting blood samples were collected from all enrolled subjects. All blood biochemical measurements, including blood urea nitrogen (BUN), creatinine (CR), triglycerides (TGs), LDL-C, HDL-C, apoA-I, and apolipoprotein B (apoB), were determined by an automated analyzer (AU5800 Beckman Coulter, Beckman Coulter Inc., USA), and thyroid function was analyzed by a fully automatic immunoassay analyzer (Dxl 800 Beckman Coulter, Beckman Coulter Inc., USA). Anthropometric measurements were recorded using SPSS 20.0. Baseline data were extracted from the hospital information system, such as age, sex, body weight, and height.

BMI was calculated from the values of weight divided by height squared (kg/m^2^). Pulse pressure (PP) was defined as the difference between the SBP and DBP.

### Statistical analysis

Categorical variables were expressed as frequencies and percentages and were compared using the chi-square test. Continuous variables were tested for normality with Kolmogorov–Smirnov statistics. Skew distribution variables are presented as the medians (interquartile ranges), and differences in variables among groups were analyzed using the Kruskal–Wallis H test. In the binary logistic regression, patients were classified into two groups according to the HDL-C/apoA-I ratio and FT3 level using the median as a cutoff point. Multiple linear regression analysis was conducted. Logistic regression analyses were performed to estimate the interaction of the HDL-C/apoA-I ratio and FT3 level with the risk of CAD. A value of *P* < 0.05 was considered significant. Statistical Package for the Social Sciences software version 20.0 (SPSS Inc., Chicago, IL, USA) was used for statistical analysis.

## Results

### Baseline and biochemical characteristics in patients

Table 1 displays the patients’ baseline and biochemical characteristics. Participants were divided into three groups based on the HDL-C/apoA-I ratio tertiles. Age, BMI, PP, TGs, LDL-C, apoB, FT3, TSH, and the prevalence of DM, EH and CAD were different among the three groups (*P* < 0.05). The TG values gradually decreased with the elevated HDL-C/apoA-I ratio tertiles. Patients in tertile 2 had higher LDL-C and apoB levels than those in tertiles 1 and 3. Subjects in the highest HDL-C/apoA-I ratio tertile had lower FT3 levels (4.36 pmol/L vs. 4.73 pmol/L, *P* < 0.001), TSH levels (1.69 mIU/L vs. 2.02 mIU/L, *P* < 0.001) and CAD (71% vs. 79%, *P* = 0.204 as indicated in Table 1). There was no difference in apoB and TSH between the low and middle levels of the HDL-C/apoA-I ratio. The prevalence of CAD in the tertile 3 group was significantly lower than that in the tertile 2 group (*P* = 0.006).

**Table 1:** Baseline and biochemical characteristics in patients

Variable	HDL-C/apoA-I ( mmol/g)							
	Total (n = 1686)	T1 (0.190–0.836)	T2 (0.837–0.947)	T3 (0.948–5.500)	*P*	*P*1	*P*2	*P*3
		(n = 563)	(n = 558)	(n = 565)				
Age, years	58.0 (66.0–72.0)	64.0 (56.0–71.0)	66.0 (59.0–72.0)	68.0 (60.0–73.5)	< 0.001	0.020	< 0.001	0.023
Male [n (%)]	1115 (66.1)	384 (68.2)	352 (63.0)	379 (67.0)	0.163			
BMI (kg/m^2^)	24.6 (22.5–26.9)	25.3 (23.4–27.4)	24.7 (22.8–26.0)	23.4 (21.5–25.7)	< 0.001	0.010	< 0.001	< 0.001
PP (mm/Hg)	55.0 (45.0–66.0)	55.0 (47.0–65.0)	55.0 (46.0–68.0)	54.0 (44.0–64.0)	0.027	1.000	0.157	0.030
BUN/CR (g/mmol)	0.78 (0.65–0.95)	0.08 (0.06–0.95)	0.08 (0.07–0.96)	0.08 (0.06–0.94)	0.156			
TGs (mmol/L)	1.50 (1.08–2.11)	2.05 (1.48–3.09)	1.53 (1.17–2.00)	1.11 (0.86–1.46)	< 0.001	< 0.001	< 0.001	< 0.001
LDL-C (mmol/L)	2.63 (2.05–3.29))	2.52 (2.02–3.09)	2.85 (2.21–3.45)	2.62 (1.97–3.39)	< 0.001	< 0.001	0.046	0.024
ApoB (g/L)	0.84 (0.67–1.04)	0.85 (0.70–1.11)	0.88 (0.70–1.04)	0.78 (0.62–0.95)	< 0.001	1.000	< 0.001	< 0.001
FT3 (pmol/L)	4.51 (4.06–5.02)	4.73 (4.29–5.20)	4.46 (4.05–4.95)	4.36 (3.84–4.86)	< 0.001	< 0.001	< 0.001	0.025
FT4 ( pmol/L)	16.85 (15.06–18.79)	16.98 (14.99–18.73)	16.52 (14.98–18.40)	16.97 (15.26–19.11)	0.065			
TSH (mIU/L)	1.85 (1.18–2.96)	2.02 (1.24–3.17)	1.94 (1.27–3.00)	1.69 (1.08–2.65)	< 0.001	1.000	< 0.001	0.001
DM, n (%)	1279 (75.8)	179 (32.0)	144 (25.0)	105 (19.0)	< 0.001	0.049	< 0.001	0.018
EH, n (%)	1170 (69.3)	421 (74.0)	384 (69.0)	365 (64.0)	0.001	0.090	0.001	0.406
CAD, n (%)	1279 (75.8)	448 (79.0)	426 (76.0)	405 (71.0)	0.008	0.620	0.204	0.006

*P*1, T1 versus T2; *P*2, T1 versus T3; *P*3, T2 versus T3

*BMI* Body mass index, *PP* pulse pressure, *DM* Diabetes mellitus, *EH* essential hypertension, *BUN* blood urea nitrogen, *CR* creatinine, *TGs* triglycerides, *LDL-C* low-density lipoprotein cholesterol, *ApoB* apolipoprotein B, *apoA-I* apolipoprotein A-I, *HDL-C* high-density lipoprotein cholesterol, *FT3* free triiodothyronine, *FT4* free thyroxine, *TSH* thyroid stimulating hormone, *CAD* coronary artery disease, *T* tertile

### Multiple linear regression analysis for the association between the HDL-C/apoA-I ratio and FT3 level

The HDL-C/apoA-I ratio was used as the dependent variable, and age, sex, BMI, TGs, LDL-C, ApoB, FT3, FT4 and TSH were used as independent variables in multiple linear regression analysis. Table 2 shows that FT3and TSH levels were negatively associated with the HDL-C/apoA-I (*P* < 0.005). When FT3 levels and TSH levels increased by 1 pmol/L, HDL-C/apoA-I reduced by 0.116 and 0.061 mmol/g, respectively. There was a negative correlation between TGs, BMI and the HDL-C/apoA-I ratio (*P* < 0.005). The HDL-C/apoA-I ratio decreased by 0.506 mmol/g for every 1 mmol/L increase in the TG level. LDL-C was positively correlated with the HDL-C/apoA-I ratio. On average, the HDL-C/apoA-I ratio increased by 0.051 mmol/g for every 1 mmol/L increase in the LDL-C level.

**Table 2:** Multiple linear regression analysis for the association between the HDL-C/apoA-I ratio and FT3 level

Variable	SE	Coef	T	*P*
Age	0.000	0.013	0.568	0.570
Sex	0.007	0.020	0.888	0.375
BMI	0.000	− 0.086	− 3.985	< 0.001
TGs	0.002	− 0.506	− 19.44	< 0.001
LDL-C	0.000	0.051	2.369	0.018
ApoB	0.013	0.028	1.078	0.281
FT3	0.002	− 0.116	− 5.252	< 0.001
FT4	0.001	0.002	0.088	0.930
TSH	0.001	− 0.061	− 2.744	0.006

*BMI* Body mass index, *TGs* Triglycerides, *LDL-C* low-density lipoprotein cholesterol, *ApoB* Apolipoprotein B, *FT3* free triiodothyronine, *FT4* free thyroxine, *TSH* thyroid-stimulating hormone

### Logistic regression analysis of the risk of CAD with the HDL-C/apoA-I ratio and FT3 level

The HDL-C/apoA-I ratio and FT3 level were divided into two groups as medians. Logistic regression analyses were used to explore the association of the HDL-C/apoA-I ratio and FT3 level with the risk of CAD (Table 3). With the lower median group as the reference, we found that the risk of CAD was significantly lower in the group with a higher HDL-C/apoA-I ratio and FT3 level.

**Table 3:** Logistic regression analysis of the risk of CAD with the HDL-C/apoA-I ratio and FT3 level

Variable		*P*	*OR* (95% CI)
FT3	≤ 4.5 pmol/L		Reference
FT3	> 4.5 pmol/L	0.000	0.658 (0.523–0.827)
HDL-C/apoA-I	≤ 0.89 mmol/g		Reference
HDL-C/apoA-I	> 0.89 mmol/g	0.000	0.614 (0.488–0.772)

*FT3* Free triiodothyronine, *HDL-C* high-density lipoprotein cholesterol, *apoA-I* apolipoprotein A-I, *OR* odds ratio, *CI*: confidence internal

**Fig. 2:**
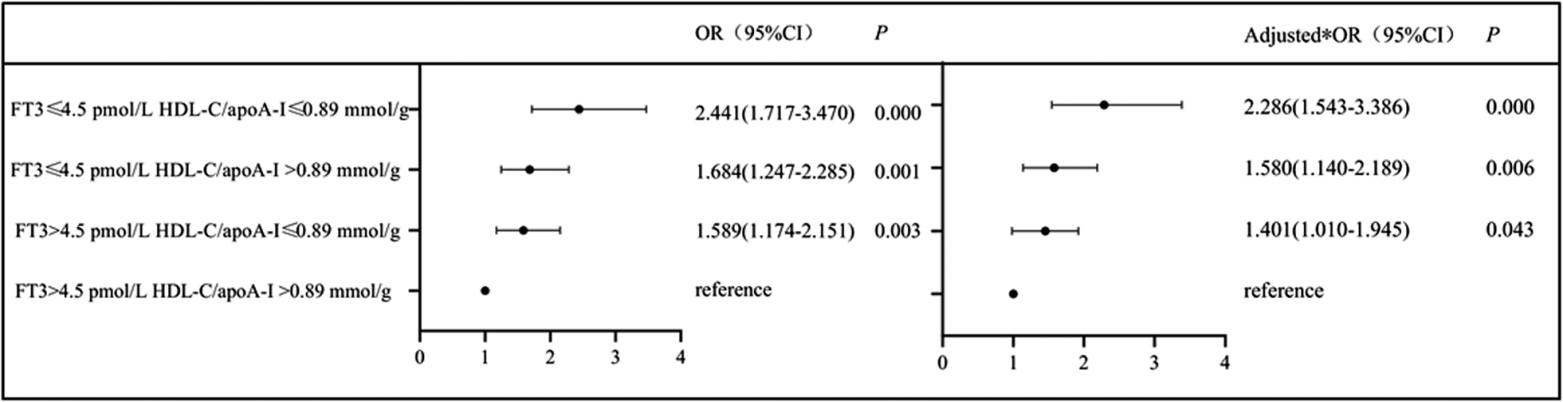
Interaction of the HDL-C/apoA-I ratio and FT3 level with the risk of CAD using binary logistic regression. *Adjusted for age, sex, BMI, EH, LDL-C and TGs

To assess the interaction of the HDL-C/apoA-I ratio and FT3 level with the risk of CAD, participants were divided into four groups (Group 1: FT3 > 4.5 pmol/L and HDL-C/apoA-I > 0.89 mmol/g; Group 2: FT3 > 4.5 pmol/L and HDL-C/apoA-I ≤ 0.89 mmol/g; Group 3: FT3 ≤ 4.5 pmol/L and HDL-C/apoA-I > 0.89 mmol/g; Group 4: FT3 ≤ 4.5 pmol/L and HDL-C/apoA-I ≤ 0.89 mmol/g). Odds ratio (OR) and *P* values are shown in Fig. 2. Taking Group 1 as a reference, the patients in Group 4 were associated with the highest risk of CAD (OR = 2.441, 95% CI = 1.717–3.470), adjusted for age, sex, BMI, EH, LDL-C and TGs (OR = 2.286, 95% CI = 1.543–3.386). Also, the adjusted risk of CAD was higher for patients with FT3 ≤ 4.5 pmol/L (Group 3) than for those with a low HDL-C/apoA-I ≤ 0.89 mmol/g (Group 2), as Fig. 2 shows.

### Stratified analysis of the interaction between the HDL-C/apoA-I ratio and FT3 level

**Fig. 3:**
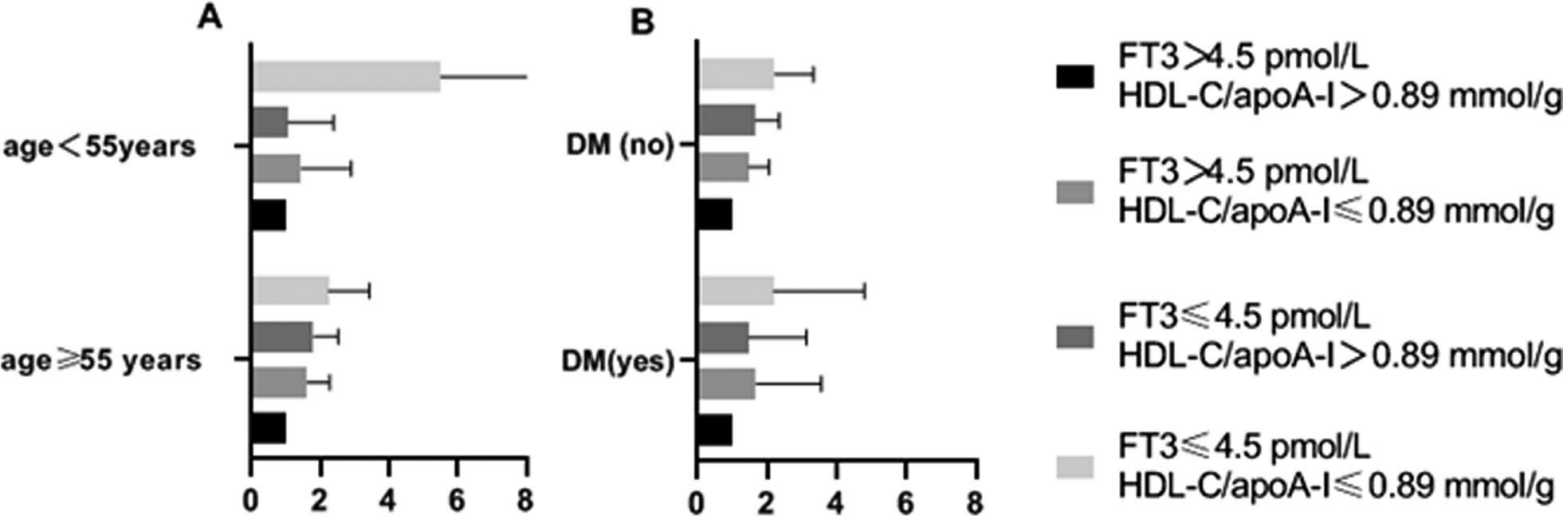
Stratified analysis of the interaction between the HDL-C/apoA-I ratio and FT3 level by age, DM status

The HDL-C/apoA-I- FT3 and stratified factors included age, sex, BMI, EH status and DM status on the risk of CAD are shown in Table 4. The HDL/apoA-I-FT3-stratified risk factor interactions revealed that CAD risk in age < 55 years or non-DM with HDL-C/apoA-I ≤ 0.89 mmol/g and FT3 ≤ 4.5 pmol/L was stronger than those age ≥ 55 years or DM (Fig. 3A and B). HDL-C/apoA-I ≤ 0.89 mmol/g showed significant interactions with FT3 ≤ 4.5 pmol/L on CAD risk in patients with hypertension (OR = 2.446, 95% CI = 1.570–3.813). The interaction between HDL-C/apoA-I ≤ 0.89 mmol/g and FT3 > 4.5 pmol/L on the risk of CAD was the strongest in obese patients (OR = 2.966, 95% CI = 1.374–6.405).

**Table 4:** Stratified analysis of the interaction between the HDL-C/apoA-I ratio and FT3 level

Variable	HDL-C/apoA-I			
	> 0.89 mmol/g		≤ 0.89 mmol/g	
	OR (95% CI)	*P*	OR (95% CI)	*P*
FT3 > 4.5 pmol/L *age ≥ 55 years			1.613 (1.150–2.262)	0.006
FT3 > 4.5 pmol/L *age < 55 years			1.445 (0.722–2.892)	0.298
FT3 ≤ 4.5 pmol/L*age5 ≥ 55 years	1.805 (1.298–2.511)	< 0.001	2.231 (1.534–3.245)	0.002
FT3 ≤ 4.5 pmol/L *age < 55 years	1.049 (0.461–2.388)	0.910	5.506 (1.741–17.408)	0.004
FT3 > 4.5 pmol/L *male			1.748 (1.199–2.547)	0.004
FT3 > 4.5 pmol/L *female			1.129 (0.661–1.927)	0.658
FT3 ≤ 4.5 pmol/L*male	1.543 (1.041–2.287)	0.031	2.578 (1.591–4.177)	< 0.001
FT3 ≤ 4.5 pmol/L*female	2.198 (1.337–3.614)	0.002	2.803 (1.633–4.809)	< 0.001
FT3 > 4.5 pmol/L *BMI ≥ 28 kg/m^2^			2.966 (1.374–6.405)	0.006
FT3 > 4.5 pmol/L *BMI28 < kg/m^2^			1.381 (0.981–1.944)	0.065
FT3 ≤ 4.5 pmol/L* BMI ≥ 28 kg/m^2^	3.062 (1.235–7.592)	0.016	2.923 (1.223–6.988)	0.016
FT3 ≤ 4.5 pmol/L* BMI < 28 kg/m^2^	1.469 (1.049–2.059)	0.025	2.333 (1.561–3.489)	< 0.001
FT3 > 4.5 pmol/L*EH (yes)			1.420 (0.974–2.270)	0.069
FT3 > 4.5 pmol/L*EH (no)			1.790 (1.063–3.014)	0.029
FT3 ≤ 4.5 pmol/L*EH (yes)	1.555 (1.056–2.292)	0.025	2.446 (1.570–3.813)	< 0.001
FT3 ≤ 4.5 pmol/L*EH (no)	1.850 (1.118–3.061)	0.017	2.273 (1.252–4.125)	0.007
FT3 > 4.5 pmol/L* DM (yes)			1.683 (0.797–3.552)	0.172
FT3 > 4.5 pmol/L* DM (no)			1.469 (1.049–2.056)	0.025
FT3 ≤ 4.5 pmol/L* DM (yes)	1.476 (0.697–3.127)	0.309	2.184 (0.994–4.796)	0.052
FT3 ≤ 4.5 pmol/L* DM (no)	1.663 (1.186–2.332)	0.003	2.229 (1.493–3.328)	< 0.001

*BMI* Body mass index, *EH* essential hypertension, *DM* diabetic mellitus, *HDL-C* high-density lipoprotein cholesterol, *apoA-I* apolipoprotein A-I, *FT3* free triiodothyronine

## Discussion

The present study showed that low HDL-C/apoA-I and low FT3 level could increase CAD risk, which was similar to previous studies [17, 18]. In addition, we first found an interactive role in the association between the HDL-C/apoA-I ratio and FT3 level. The risk of CAD was significantly increased in subjects with HDL-C/apoA-I ≤ 0.89 mmol/g and FT3 ≤ 4.5 pmol/L.

The concentrations of HDL-C and apoA-I were strongly and inversely associated with the risk of CAD in many studies [19]. Previous studies found that every 1 mg (0.03 mmol/L) increase in HDL-C reduced the risk of future CAD by 2–3%. However, other studies showed that increasing the HDL-C level by inhibiting the cholesterol ester transfer protein (CETP) failed to decrease cardiovascular events [3]. The Atherothrombosis Intervention in Metabolic Syndrome with Low HDL/High Triglycerides: Impact on Global Health Outcomes (AIM-HIGH) enrolled 3414 patients and showed no clinical benefit from the addition of niacin during a 36-month follow-up period [20]. Group AS et al. also failed to reduce cardiovascular disease by increasing HDL-C with fibrates [21]. So, more and more researchers have paid more attention to HDL size, which may be associated with cardiovascular disease and diabetes. Norman A et al. proposed that the HDL-C/apoA-I ratio could be an available biomarker for estimating HDL size by a large-scale experimental examination of the updated Shen model [9].

The HDL-C/apoA-I ratio is a biomarker of HDL-C particles to predict cardiovascular risk [22]. A previous study showed that oxidative damage to HDL particle may decrease its capacity to promote cholesterol efflux [23]. Miller et al. found that there was a lower HDL-C/apoA-I ratio in patients with CAD, suggesting that a lower HDL-C/apoA-I ratio might be associated with higher cardiovascular risk [24]. However, some studies pointed out that increased HDL-C/apoA-I ratios were associated with higher coronary artery calcium scores, risk of CAD, subclinical atherosclerosis and mortality [25]. In the present study, we found that the prevalence of CAD was significantly highest in the lowest HDL-C/apoA-I ratio tertile. Logistic regression analysis further showed that the HDL-C/apoA-I ratio was a protective factor for CAD (OR = 0.614, 95% CI = 0.488–0.772, *P* = 0.000).

Thyroid dysfunction was found in 23.3% of patients with CAD [26]. Hypothyroidism is known to increase LDL-C, TGs and HDL-C [27], which is possibly due to the reduction in catabolism of lipoproteins. Patients with subclinical hypothyroidism have higher levels of inflammatory markers, which can promote CAD [28]. Thyroid hormone has direct anti-atherosclerotic effects, such as nitric oxide production and suppression of smooth muscle cell proliferation [29]. Coceani et al. showed that the FT3 levels were inversely related to CAD presence [30]. In a study enrolling 588 outpatients with suspected CAD, FT3 levels were inversely associated with artery calcification scores and the incidence of major adverse cardiac events [31].

Hypothyroidism is known to be associated with lipid disturbances and abnormal plasma protein levels [32, 33]. In some studies changes in the composition and size of plasma HDL-C were observed in patients with hypothyroidism [34, 35]. Anna et al. pointed out that CETP and phospholipid transfer protein (PLTP) activity were decreased in patients with hypothyroidism which was associated with decreased HDL2 and increased HDL3 cholesterol levels [28]. ApoA is the main component of HDL, which is related to the function of HDL [36]. Treatment with T3 caused a decrease in HDL particle size and an increase in lipid-poor apoA-I in hypophysectomized rats and T3 enhanced the ability of serum to accept cellular cholesterol by the ABCA1 transporter [37].

The results of multiple linear regression analysis showed that FT3 and TSH levels were independent predictors for the HDL-C/apoA-I ratio and negatively associated with the HDL-C/apoA-I ratio(*P* < 0.005). With FT3 and TSH increasing by 1 pmol/L, the HDL-C/apoA-I ratio decreased by 0.116 and 0.061 mmol/g, respectively. A previous study showed that T3 might increase the mRNA levels of CYP7A1, which is a key enzyme in the conversion of cholesterol to bile acids for excretion into the bile [38, 39]. T3 might increase the scavenger receptor-BI protein levels of the liver, leading to decreased HDL-C levels [38].

This was the first study to investigate the interaction between the HDL-C/apoA-I ratio and the FT3 level with the risk of CAD. The main findings showed that patients in the lower median for the HDL-C/apoA-I ratio (≤ 0.89 mmol/g) and FT3 level (≤ 4.5 pmol/L) had the highest CAD risk (OR = 2.441, 95% CI = 1.717–3.470, *P* < 0. 001).

HDL-C/apoA-I-FT3-stratified risk factor interaction was analyzed in our study. The association of lower age group, female, EH, non-DM with CAD risk was the strongest in patients with the HDL-C/apoA-I ratio (≤ 0.89 mmol/g) and FT3 level (≤ 4.5 pmol/L). This may guide us to evaluate the risk of CAD in clinical work, and the specific mechanism needs further study.

## Limitations

Several limitations existed in the present study. First, the present study was a hospital-based observation study. The sample size was small and the number of cases and controls was not absolutely matched. Second, we could not analyze some useful data, such as C-reactive protein. Third, we could not obtain some useful data, such as medication records. Fourth, we did not perform optical coherence tomography (OCT) or vascular ultrasound.

## Conclusion

The HDL-C/apoA-I ratio was negatively associated with the FT3 level, and there was a significant interaction between the HDL-C/apoA-I ratio and FT3 level with the risk of CAD.

## Data Availability

All relevant data and materials are included in the manuscript. The datasets will be available from the corresponding author on reasonable requests after study completion.

## Abbreviations

BMI: Body mass index
PP: Pulse pressure
DM: Diabetes mellitus
EH: Essential hypertension
BUN: Blood urea nitrogen
CR: Creatinine
TGs: Triglycerides
LDL-C: Low-density lipoprotein cholesterol
ApoB: Apolipoprotein B
apoA-I: Apolipoprotein A-I
HDL-C: High-density lipoprotein cholesterol
FT3: Free triiodothyronine
FT4: Free thyroxine
TSH: Thyroid stimulating hormone
CAD: Coronary artery disease
OR: Odds ratio
CI: Confidence interval
